# Volume of Blood

**Published:** 1915-10

**Authors:** 


**Volume of Blood.** L. G. Rowntree, N. Keith, and J. T. Geraghty, of Baltimore, in a paper before the Association of American Physicians, report the function of a non-toxic, non-dialysable red dye, with estimation of its dilution after 3 to 6 minutes when it should have been evenly distributed. By this calculation, they estimate the normal blood volume at 85 c.c. per kilo, or 12 per cent.

